# Case Report: Pregnancy in a patient with recurrent glioblastoma

**DOI:** 10.12688/f1000research.2-246.v1

**Published:** 2013-11-15

**Authors:** Birgit Flechl, Marco Ronald Hassler, Gerhard Kopetzky, Peter Balcke, Christine Kurz, Christine Marosi

**Affiliations:** 1 Department of Internal Medicine I, Clinical Division of Oncology, Medical University of Vienna, 1090 Vienna, Austria; 2 Department of Internal Medicine I, General Hospital of St. Pölten, 3100 St. Pölten, Austria; 3 Department of Obstetrics and Gynaecology, Clinical Division of Endocrinology, Medical University of Vienna, 1090 Vienna, Austria; 4 Comprehensive Cancer Center-Central Nervous System Tumours Unit (CCC-CNS), Medical University of Vienna, 1090 Vienna, Austria

## Abstract

We report the case of a woman with relapsed glioblastoma multiforme (GBM) who recently gave birth. She announced her pregnancy shortly after the sixth cycle of a dense regimen of temozolomide, prescribed for treating the first recurrence of glioblastoma. Three years ago, in April 2008, she had undergone gross total resection of a glioblastoma multiforme in the postcentral region of the right hemisphere and had subsequently received treatment according to the actual standard therapy consisting of radiotherapy up to 60 Gy with concomitant and adjuvant temozolomide. The complete amount of temozolomide given before this pregnancy was 20.9 mg/m ^2^. Nevertheless, she delivered a 1890 g child by caesarean section in the 32/6 week of pregnancy. The child showed no anomalies and is developing normally under close surveillance by paediatricians.

## Introduction

Cases of pregnancy and giving birth are rare in patients with malignant gliomas. Besides the grim prognosis of malignant gliomas, the actual standard therapy of glioblastoma adversely affects fertility ^1^. In fact, radiotherapy aimed at the hypothalamus and the pituitary gland exceeding 45 Gy could impair the synthesis of gonadotropins, while chemotherapy with alkylating agents such as temozolomide is associated with impaired ovarian function leading to premature menopause ^1– 3^. However, there are some reports of pregnancies in women with malignant gliomas ^1, 4– 11^, describing women whose brain tumors were diagnosed during pregnancy, as well as women who already had undergone treatment for malignant gliomas and who became pregnant afterwards. Our recent patient is an example of a patient whose fertility was not suppressed by glioblastoma multiforme (GBM) and its treatment.

## Case

The patient is a 37 year old woman, born in Austria. She had two children (13 and 15 years) before her GBM diagnosis. She was diagnosed with GBM in the right frontal lobe in April 2008, and treated according to a current treatment standard consisting of a gross total resection of the tumor, fractionated confocal radiotherapy up to 60 Gy (2 Gy/fraction) and concomitant and adjuvant chemotherapy with temozolomide ^12^. Diagnosis and treatment were performed at the Medical University of Vienna (MUV). Following the concomitant therapy, she experienced amenorrhea for 6 months, followed by irregular menstrual cycles with oligomenorrhea. Two years later, a local recurrence of GBM was diagnosed. She underwent a second resection and followed 6 cycles of dose dense temozolomide, 100 mg/m ^2^ orally for five days per week, with a drug holiday over every weekend. She became pregnant less than three weeks after the last intake of the sixth cycle of temozolomide, after a total dose of temozolomide of 20.9 g/m ^2^ ( Table 1).

**Table 1.** Total temozolomide dose before the pregnancy.

	Temozolomide scheme	Temozolomide dose
**Concomitant phase**	42 days at 75 mg/m ^2^	3150 mg/m ^2^
**Adjuvant treatment**	5× 150 mg/m ^2^+ 5× 5×200 mg/m ^2^	5750 mg/m ^2^
**Dose 1 ^st^ line treatment**	-	8900 mg/m ^2^
**Dose temozolomide** **relapse**	6× 100 mg/m ^2^ × 5 days × 4 weeks/month	12000 mg/m ^2^
**Total dose**		20.900 mg/m ^2^

Both parents wanted to carry the child to term. They were offered intensified pregnancy monitoring and genetic counselling, but no genetic tests were performed. During pregnancy the child developed normally as followed by close meshed ultrasound controls and the mother was doing well until week 27, when she developed signs of increased intracranial pressure as well as weakness of the left leg. She was admitted to the local general hospital for observance and anti edematous treatment. As the mother’s condition worsened, she received corticosteroids to induce lung maturation of the fetus, and the child was delivered after 32 weeks and 6 days of pregnancy by caesarean section using the Misgav Ladach method ^13, 14^. The child showed Apgar scores of 8/9/9, one, five and ten minutes after birth, respectively, weighed 1876 g, cried spontaneously and was neurologically inconspicuous. The infant did not need further respiratory assistance after the third day of life, and was discharged from the neonatal intensive care unit after an uncomplicated stay without any signs of neurological or any other organ deficit, weighing 2.5 kg. The child has shown normal growth and development ever since.

The mother underwent her third neurosurgical procedure two weeks after delivery, followed by chemotherapy with fotemustine 100 mg/m ^2^ every three weeks for six cycles combined with bevacizumab 10 mg/kg every two weeks 6 weeks later. Eight months after the caesarean section, distal progression in the brain was diagnosed on MRI and she was referred for radiotherapy of the distant recurrence.

## Discussion

Temozolomide has been classified to pregnancy category D by the US Food and Drug Administration, which means that animal studies have revealed evidence of embryo lethality induced by the drug. Moreover, malformations have been observed in animals, therefore women are advised to avoid becoming pregnant during temozolomide intake. However, we report here the pregnancy of a woman with GBM, treated with radio/chemotherapy using a protocol described by Stupp *et al.* ^12^ and further dose dense temozolomide in relapse 5 days of seven, as described by Strik *et al.* ^15^.

There are some previous reports on pregnancies in women with malignant glioma. Nadine Johnson retrospectively reported the obstetric outcomes of women with intracranial neoplasms, from Ontario, Canada, covering the experience of 6000 deliveries. In this database, Johnson *et al.* identified 25 pregnancies in women who had been diagnosed with intracranial neoplasms, including 12 patients with gliomas ^8^. This review discusses neurological deterioration due to the hormonal changes causing fluid retention during pregnancy and the complications and the management of increased cranial pressure at the time of delivery. There are some small series and case reports on the same issues ^4, 7– 10, 14, 16, 17^.

Deborah Blumenthal (2008) reported a series of 6 women with malignant glioma with unplanned pregnancies during glioma treatment. Several women even had drug exposure during the first trimester of pregnancy, three with PCV (procarbazine, cyclophosphamide, vincristine), and three with temozolomide ^17^. All gave birth to healthy, full term infants with no evidence of congenital malformations. Sadly, five of the six mothers died within the following 2.5 years due to recurrence of disease ^17^.

An adverse effect on disease outcome during pregnancy has been observed in six of eight pregnancies in women with low grade gliomas followed by the French low grade glioma (LGG) group, possibly due to the decreased immune surveillance during pregnancy or to the presence of potential hormonal receptors on glioma cells ^4, 16^. In a later series on 12 pregnancies of women with LGG followed by the same research group published 2010, the growth rate of the gliomas increased during pregnancy as measured by successive MRI scans ^16^.

However, the prolonged survival of our patient 18+ months after the diagnosis of a relapsed glioblastoma appears noteworthy. This case also should bring to mind the necessity of repeated counselling about contraception – even in patients where the probability of persistent fertility is minimal, as in the patient described in this report. In fact, regardless of all the apprehensions of medical professionals about this pregnancy, the patient and her husband were simply happy about it. They wanted to keep this child, even if it would have meant severe adverse complications for the mother. They perceived this pregnancy as a gift of life and enjoyed every moment, even when it was steadily overshadowed by the glioblastoma. They remembered perfectly well that they had been told that pregnancy should be avoided during and after the glioma treatment, and that they even had signed this as a part of the regular consent for treatment; but on the other hand they told us that this pregnancy had been the only happy event for them in all those years.

They also tried to organize further care for the child, as the possibility of the baby losing its mother during childhood is very high. As the patient and her husband are aware of the grim prognosis of recurrent GBM, they planned to raise the child with multiple psychological parents, including its grown up siblings and the two sisters of the mother.

## Conclusion

This report shows that pregnancy and the birth of a healthy infant can occur even in women that have been heavily pretreated with alkylating agents and that conception can occur as early as three weeks after the last intake of chemotherapy.

## Consent

Written informed consent was obtained from the patient for the publication of this case report.
