# Supplementary material for: Case Report: Pregnancy in a patient with recurrent glioblastoma
Source: F1000Res. 2013 Nov 15;2:246. [Version 1] doi: 10.12688/f1000research.2-246.v1 (PMC4103487; doi:10.12688/f1000research.2-246.v1)
Supplement: Peer review history

# Peer review: Referee response for version 1

**DOI:** 10.5256/f1000research.2872.r4214

**Published:** 2014-03-21

**Authors:** Mari-Paule Thiet

**Affiliations:** 1 Departments of Obstetrics, Gynecology, and Reproductive Sciences, University of California, San Francisco, CA, USA

This article describes a single case of pregnancy following a diagnosis and treatment of recurrent glioblastoma. It illustrates that spontaneous pregnancy can be achieved after high doses of chemotherapy. There is a review of the current literature regarding outcomes of pregnancy in patients with brain tumors.

I have read this submission. I believe that I have an appropriate level of expertise to confirm that it is of an acceptable scientific standard.

# Peer review: Referee response for version 1

**DOI:** 10.5256/f1000research.2872.r3122

**Published:** 2014-01-14

**Authors:** Deborah Blumenthal

**Affiliations:** 1 Oncology Division, Tel-Aviv Sourasky Medical Center, Tel-Aviv, Israel

The report, " *Pregnancy in a patient with recurrent glioblastoma*" illustrates a notable case of a 37 year-old woman with recurrent glioblastoma (GB)  who became pregnant within 3 weeks of a (6–cycle) dose-dense temozolomide regimen. The case is notable for preservation of fertility, essentially during alkylating treatment; positive outcome of the child at planned 32 week delivery by C-section and normal growth and development at 18 months; and for survival of the mother with recurrent GB 18+ months from recurrence.

The authors mention prior related works in the literature regarding the effects of pregnancy on patients with malignant glioma; a series of patients who conceived (with positive fetal outcome) during active alkylator treatment and documentation of accelerated growth rates of gliomas during pregnancy.

This report is encouraging for those patients concerned about fertility issues, but should not be interpreted as a guarantee for either male or female patients that fertility will be preserved after alkylating chemotherapy. Additionally, a disclaimer should be made for the potential (confirmed by animal studies on embryo-lethality with exposure to these drugs) danger to the exposed fetus, despite the reported good outcomes of these children.

I have read this submission. I believe that I have an appropriate level of expertise to confirm that it is of an acceptable scientific standard.
